# Retraction Note: Clinicopathologic characterization of visceral gout of various internal organs – a study of 2 cases from a venom and toxin research center

**DOI:** 10.1186/s13000-016-0569-0

**Published:** 2016-11-02

**Authors:** Alireza Nasoori, Behnam Pedram, Zahra Kamyabi-Moghaddam, Aram Mokarizadeh, Hamid Pirasteh, Amir Farshid Fayyaz, Mohammad Barat Shooshtari

**Affiliations:** 1 Biotechnology Research Center, Department of Medical Biotechnology, Venom and Toxin Unit, Pasteur Institute of Iran, Tehran, Iran; 2 Department of Pathobiology, Susangerd Branch, Islamic Azad University, Susangerd, Iran; 3 Department of Medicine II, Klinikum rechts der Isar, TU Muenchen, Munich, Germany; 4 Department of Pathology, Faculty of Veterinary Medicines, Tehran University, Tehran, Iran; 5 Cellular and Molecular Research Center, Kurdistan University of Medical Sciences, Sanandaj, Iran; 6 Department of Nephrology, AJA University of Medical Sciences, Tehran, Iran; 7 Department of Legal Medicine, AJA University of Medical Science, Tehran, Iran; 8 Biotechnology Research Center, Science and Technology Institute, Tehran, Iran

## Retraction

The Editor-in-Chief and Publisher have retracted this article [1] because the scientific integrity of the content cannot be guaranteed. An investigation by the Publisher found it to be one of a group of articles we have identified as showing evidence suggestive of attempts to subvert the peer review and publication system to inappropriately obtain or allocate authorship. This article showed evidence of peer review and authorship manipulation.
